# Qualitative analysis of first-person accounts of noetic experiences

**DOI:** 10.12688/f1000research.52957.1

**Published:** 2021-06-25

**Authors:** Helané Wahbeh, Nina Fry, Paolo Speirn, Lutvija Hrnjic, Emma Ancel, Erica Niebauer

**Affiliations:** 1 Research, Institute of Noetic Sciences, Petaluma, CA, 94928, USA; 2 Neurology, Oregon Health & Science University, Portland, OR, 97239, USA

**Keywords:** noetic, noetic experiences, subjective, qualitative, psychology, intuition, parapsychology

## Abstract

The term “noetic” comes from the Greek word noēsis/noētikos that means inner wisdom, direct knowing, intuition, or implicit understanding. Strong cultural taboos exist about sharing these experiences. Thus, many may not feel comfortable transparently discussing or researching these topics, despite growing evidence that these experiences may be real. The study’s objective was to qualitatively evaluate first-hand accounts of noetic experiences. 521 English-speaking adults from around the world completed an online survey that collected demographic data and four open-ended questions about noetic experiences. Thematic analysis was used to characterize the data. The ten most used codes were expressing to or sharing with others, impacting decision-making, intuition/”just knowing,” meditation/hypnosis, inner visions, setting intentions/getting into the “state,” healing others, writing for self, and inner voice. There were five main themes identified: 1. Ways of Engagement; 2. Ways of Knowing; 3. Types of Information; 4. Ways of Affecting; and 5. Ways of Expressing. Subthemes. Future research will include investigating the nuances of these themes and also establishing standardized methods for evaluating them. This would also then inform curricula and therapies to support people in these experiences.

## Introduction

The term “Noetic” comes from the Greek word noēsis/noētikos that means inner wisdom, direct knowing, intuition, or implicit understanding. William James, American philosopher and psychologist, defined noetic experiences as “states of knowledge. They are states of insight into depths of truth unplumbed by the discursive intellect. They are illuminations, revelations, full of significance and importance, all inarticulate though they remain; and as a rule, they carry with them a curious sense of authority for after-time” ( James, 1985, pp. 380–381).

William James refers to the phenomenon that noetic experiences often feel like a state of understanding intuitively accessed knowledge, known as truth. One arrives at this state without intellectual, left-brain analysis. The experience is also ineffable in that the experience is hard to describe in words. These noetic experiences are present in the oldest of humanity's written records in cultures worldwide ( Hastings, 1991; Klimo, 1998).

Many words have been ascribed to the noetic experience: intuition; clairvoyance; telepathy; psychokinesis; precognition; psi; psychic; extended human capacities; and anomalous information reception, to name a few. Strong taboos preclude open discussion of these topics in most Western academic settings ( Cardeña, 2015; Schooler *et al.*, 2018; Sidky, 2018). Thus, many may not feel comfortable transparently discussing or researching these topics, despite growing evidence for them in laboratories and real-world settings ( Cardeña, 2018; Cardeña *et al.*, 2015) and their rampant global prevalence ( Bourguignon, 1976; Castro *et al.*, 2014; A. Greeley, 1987; Haraldsson, 1985, 2011; Haraldsson & Houtkooper, 1991; Hunter & Luke, 2014, pp. 101, 211, 231, 234, 237; McClenon, 1993; Palmer, 1979; Ross & Joshi, 1992).

Some propose that all people have the capacity for noetic experiences, that it is an innate human capacity. This notion is proposed in models such as the Psi-Mediated Instrumental Response (PMIR) model ( Stanford, 2015) and First-Sight Model and Theory (FSMT) ( Carpenter, 2014). The PMIR model proposes that people unconsciously access information relevant to what they need and then unconsciously use it to modify their behavior to meet their needs. The FSMT proposed that human's essential nature is to actively, continuously, and unconsciously participate in the world, which extends beyond our immediate boundaries of perceived space and time. All of our experiences and behaviors result from unconscious psychological processes that are acted out based on multiple sources of information, including those beyond our traditional five senses *.* Interestingly, cosmology and quantum physics research support this notion, with informational and holographic theories gaining support. Cosmologist Dr. Jude Currivan expresses, “experiences of nonlocal awareness that are capable of transcending space-time, while nonetheless extraordinary, should come to be seen as innate abilities” ( Currivan, 2017, p. 197).

Assuming that some noetic experiences are an innate human capacity, what is the phenomenological experience of them? Is there variation amongst people for how they are perceived and their function in people's lives? Likely, noetic experiences exist on a spectrum, from common, well-studied experiences, like empathy ( de Waal & Preston, 2017) and intuition ( Zander *et al.*, 2016) on one side and other more rare experiences, like sensing the future ( Bem *et al.*, 2016; Mossbridge & Radin, 2017) on the other side.

The goal of this research study was to qualitatively evaluate first-hand accounts of noetic experiences that go beyond our conventional notions of time and space and our traditional five senses.

## Methods

Potential participants were recruited worldwide from the Institute of Noetic Sciences (IONS) membership community, associated social networks, personal contacts and recommendations, and SurveyMonkey's target audience service. A link to an online survey administered with the SurveyMonkey platform was given to interested volunteers. The survey's first page was an informed consent form, and participants could not proceed unless they agreed. The researchers had no contact with the participants as the data was collected through the online survey. The researchers believe that noetic experiences are genuine phenomena and many have had personal noetic experiences themselves.

The inclusion criteria for participation were: 1) adults aged 18 years and older; 2) have had an experience of accessing or expressing information and energy not limited by space and time; 3) ability to understand, read, and write in English; and 4) ability to understand the consent form and complete the study activities.

Data collected from people aged 18 years or younger were destroyed. The survey collected demographic data (age, race, gender, relationship status, income, and residential location) and four open-ended questions with unlimited text limits for the response. Video and text descriptions of the context for the four questions were presented before the questions (see Supplemental Data for the survey).

The four questions were: 1) Please describe in as much detail as possible how you ACCESS INFORMATION not limited to our conventional notions of time and space (Access-Info); 2) Please describe in as much detail as possible how you ACCESS ENERGY not limited to our conventional notions of time and space (Access-Energy); 3) Please describe in as much detail as possible how you EXPRESS INFORMATION not limited to our conventional notions of time and space (Express-Info); 4) Please describe in as much detail as possible how you EXPRESS ENERGY not limited to our conventional notions of time and space (Express-Energy). The questions were separated into information and energy because of anecdotal expressions that people perceive noetic experiences as information and energy separately.

The IONS Institutional Review Board approved all study activities (IRB approval designation WAHH_2019_01).

Data was exported from SurveyMonkey into Microsoft Excel 2013 (Microsoft, Inc, Redmond, WA). Data was reviewed for valid responses and inclusion/exclusion criteria. A file with completer cases was created and then uploaded into Dedoose (version 8.3.17, Dedoose, Inc, Hermosa Beach, CA) for qualitative analysis.

Thematic analysis was used to characterize the data by grouping repeated semantic code patterns into meaningful categories/themes, as Braun and Clarke described ( Braun & Clarke, 2006). Thematic analysis was chosen because it can provide a straightforward yet rich description of participants' beliefs and experiences. Thematic analysis consists of six steps: familiarization, coding, generating themes, reviewing themes, defining and naming themes, and reporting. The approach to coding was inductive ( Saldana, 2013). The four questions were meant to elicit more comprehensive and detailed responses from the participants (rather than evaluate separate themes for each question), and thus, the responses were considered as a whole for the thematic analysis.

Four staff members performed the initial coding and applied descriptive labels generated by the data using the following steps: 1) Read through everything once becoming familiar with the data; 2) Read through everything again, this time making notes on general observations and themes, keeping the research objective in mind; 3) Read through everything again, this time generating and applying a list of broad “parent” codes; and 4) Read through everything a fourth time, this time generating and applying more specific “child” codes. After generating a more extensive, broader list of preliminary codes, they were continually refined and eventually hierarchically organized as parent and child codes. The codes and coding processes were discussed frequently among the researchers to maintain reliability across the coding process. The codes were then refined and re-organized into more detailed parent and child codes, creating a structured codebook.

The final codebook was developed with code definitions, a brief description of when and when not to use each code, and, if applicable, an example quotation. Each transcript was independently coded in duplicate using Dedoose web-based qualitative data analysis software (Dedoose, 2013). Coding was iterative, and weekly meetings were held to discuss the coding process and edit the codebook if needed. Discrepancies in coding, if any, were discussed amongst the research team, and any disagreements were resolved by consensus. An inter-rater reliability test performed before formal coding revealed excellent interrater agreement (pooled kappa score 0.84 ± 0.13) ( Cicchetti, 1994). After coding the entire dataset, the principal investigator reviewed, redistributed, and merged redundant codes. Theme summaries are provided in the results section with representative quotes for the descriptive analysis. Quotes from the data were edited with articles, punctuation, and extra clarification to support reading flow and comprehension. Numerical values for the number of participants endorsing each theme are also displayed where appropriate.

This study is reported according to the Standards for Reporting Qualitative Research ( O'Brien *et al.*, 2014).

## Results

One-thousand and thirty-three volunteers began the survey. 13 did not agree to the informed consent form, seven were under 18 years old, 450 did not enter any text, and 42 entered nonsense text. 521 people entered sensical text for at least one free-response question and were included in the analysis (Access-Info – 510, Access-Energy – 480, Express-Info – 470, Express-Energy – 442). The amount of text characters entered for each question was variable: Access-Info – 1,019 ± 1,464 (0-15,800); Access-Energy – 514 ± 724 (0-5,661); Express-Info – 400 ± 542 (0-7,878); Express-Energy – 271 ± 395 (0 – 2,798).

Participant demographics of data included in the analysis are listed in Table 1. Participants from all over the world completed the survey, with the majority of them being from the United States (364, 70.1%), followed by Canada (24, 4.6%), United Kingdom (23, 4.4%), Australia (18, 3.5%), New Zealand (7, 1.4%), South Africa (7, 1.4%), and Ireland (6, 1.2%). The following countries had five or fewer participants participating in the study: India, Belgium, Philippines, Romania, Costa Rica, Germany, Mexico, Pakistan, Portugal, Serbia, Spain, Austria, Brazil, Chile, Croatia, Finland, France, Italy, Switzerland, Argentina, China, Denmark, El Salvador, Estonia, Greece, Hungary, Indonesia, Israel, Netherlands, Nigeria, Norway, Russian Federation, Sweden, Turkey, and the United Arab Emirates.

**Table 1.** Participant demographics.

Measure	Category	Avg ± SD	%	N
**Age**	Years	52.3 ± 14.5		495
**Gender**	Female Male Other	167 347 5	66.9 32.2 1.0	519
**Race**	American Indian Asian/Pacific Islander Black or African American Hispanic White/Caucasian Other	6 21 12 31 413 33	1.1 4.1 2.3 6.0 80.0 6.4	516
**Relationship**	In a relationship Not in a relationship	254 262	46.2 50.8	516
**Income**	Under $75K $75K to $149K $150K to under $250k $250k or greater Decline to answer	258 100 44 16 97	50.1 19.4 8.5 3.1 18.8	515

Patterns emerge when we examine the entire dataset as a whole and the number of participants mentioning each concept. The top ten codes were: knowing the future (219); expressing to or sharing with others (197); impacts decision making (196); intuition/”just knowing” (184); meditation/hypnosis (183); inner visions (171); setting intentions/getting into the “state” (157); healing others (152); writing for self (151); and inner voice (151). There were five main themes identified in the data: 1. Ways of Engagement; 2. Ways of Knowing; 3. Types of Information; 4. Ways of Affecting; and 5. Ways of Expressing. Subthemes were also identified. Below, each theme is described with representative quotes from different participants.

**Theme 1: Ways of engagement**

Participants often began their responses with a description of how they engaged with the process of accessing or expressing information or energy. The ways people engaged were diverse. This theme's most typical characteristics were whether the process was intentional versus unintentional and whether the process was internally versus externally focused.

***Intentional***

Participants who mentioned specific internally-focused and intentional practices were as follows in decreasing order: meditation/hypnosis (183), setting intentions/opening/getting into the “state” (internal) (157), connecting to spirit/source/universe (126), accessing energy (117), asking (108), communing with earth (93), specific practices/preparing/ceremony (external) (89), channeling (68), professional (62), visualization (52), dreams/sleep (49), tools (pendulums, tarot cards, sigils, etc.) (42), breathwork (39), praying/prayer (39), manifestation (38), other (37), receiving training from others (32), remote viewing (30), astral projection/journeying (24), religion (23), lucid dreaming (22), traditions and rituals (18), altered states of consciousness (16), and substances/drugs (16).

People would purposefully engage in these activities to access information or energy. For example, in the following examples, participants mention meditating to access information: “When I access information on purpose, I will go into meditation;” “I meditate and will ask for information, and it will often just come; it is like an unexplainable knowing.”

Setting an intention to receive information was a common element also, in combination with the above practices or on its own. For example, “I simply get quiet, set the intention to be grounded and calm, and then start asking for assistance.” Other participant quotes were:

How I access energy by intention is I clear my mind and focus on creating space while slowing my breath to allow for energy to enter. I clear my channel and “step aside” to receive and deliver. In order to move objects, my focus must be precise, and no other thoughts can be in the way. Then I send energy outward from my mind, and the object moves.

Writing down a To-Do list also seems to call in other people to do things on my list. If I want something to occur, I declare my intention and then get as emotional as I can and repeat my desire/intention. To “let go,” laughing works best for me. It is a toss-up which is more effective, this or writing it down.

Many practices were mentioned in combination with each other rather than on their own. For example, these statements below include dreamwork, meditation, out-of-body experiences, intention setting, and breathwork.

In meditation, either sitting or lying down, I set a specific intention to either feel a specific energy/healing/sensation or to “know” specific energy/information/experience. Through relaxing my entire body, muscles, bones and settling into a slow breathing pattern, I become more “open” to experiencing my intention.

Dreams, especially when I was young. For example, if I was struggling with a difficult algebraic equation, I'd set the intention before bed that the solution would be “there” and sometimes I'd awaken remembering the act of actually working out the equation in my dream and other times, just the solution would be there. In the morning, I'd have the solution.

Another internally focused way of engagement was connecting to some force, power, or field greater than themselves. People called this force various terms, like God, Spirit, Source, Universe, Interconnected Field, Higher Consciousness, Divine, Energy, or other similar terms.

When I AM present moment awareness, I am fully connected with and not separated from “an Energy” that I call “a Power greater than myself.” Others have called it “God,” “Allah,” “Universal Energy,” “Brahman,” the “Tao,” “Jehovah,” and many other words.

I use my thoughts to reach out to Heavenly Father and my angels to seek guidance on the matters I'm considering. I hold the question in my mind with an intention to discover and feel the truth or answer to it. I push out the question and anticipate to feel what the answer or truth is for it.

Sometimes this higher power was described as outside the person with words like “connected to” or “reached out to.” Other times, participants described the connection as being within themselves. For example: “I sing affirmations while creating energetic portals (by moving my hands in these patterns) as guided by Source-within-me.”

Externally-focused and intentional practices were also mentioned, such as being in nature and shamanistic traditions and rituals, including drumming, rattles, music, and dancing. Participants also used tools such as pendulums, sigils, and tarot cards, and took substances that induce altered states of consciousness.

I access energy through opening & closing shamanic sacred space. Connect with source energy and use light rings, sound, spirit guides, power animals, drumming, rattling & smudging. Intention setting is always integrated on a journey.

Additionally, specific practices included receiving training from others like having mentors or teachers who guide them, Reiki classes, or shamanic practices. Similarly, many participants were intuitive practitioners that included a specific form of noetic practice in their professional lives, such as Reiki or other energy medicine practitioners, healing arts or mental health professionals, or psychic professionals (e.g., mediums, channelers).

### Unintentional

Many people also had unintentional, spontaneous noetic engagement experiences. These happen spontaneously during everyday life (unintentional general -79) or during altered states of consciousness (9), dissociative states (4), dreams (131), near-death or out-of-body experiences (22), and synchronicities or coincidences (56). Some people also described experiencing both intentional and unintentional experiences; that is, the two are not mutually exclusive. One person shared the following:

I experience a sense of knowing that comes spontaneously and kind of takes me by surprise. There is absolutely no connection to any other thought or information; it doesn't seem logical but always turns out to be right. Most of this kind of information is related to people where I already have so much information about them in the moment I meet them. It is like I could see right through them into their soul.

In other cases, the person is not intentionally wanting to know information about the person they are meeting; it just arrives.

My experiences have been primarily spontaneous. Not through some effort on my part. I have “heard” a voice. I have experienced information not known to me pouring in through the top of my head. I have had out-of-body experiences. I have been occupied in some way and suddenly been in a completely different state of consciousness. For instance, one time I was reading a book and suddenly seemed to be experiencing life from “Oneness” as opposed to as an individual.

Many people also mention the concept of synchronicity or coincidence in the context of gaining information that they would not usually know or as some inspiration.

It also happens with looking up at highway billboard signs. For example, there was a day I was driving to give a public talk and nervous about it. I wasn't sure I was wanted/needed by the people I was going to talk to. And, I looked up and the billboard on the highway said “Your Audience Is Waiting.”

People taking substances to achieve altered states of consciousness also spontaneously access information or energy unintentionally. Having experiences in dreams or sleep was also common. One person woke from a nightmare in which someone was stealing their bicycle keys. The next morning, they went outside to find that their bicycle had been stolen. Other people had dreams about major natural disasters, such as this participant's account of the Japanese tsunami of 2011.

I've had dreams almost all of my life, showing me events that then later actually happened and I learned about through TV and other news stations. I had a dream of me being present in, though safe from danger, as an observer watching the 2011 tsunami in Japan, three weeks before the tsunami occurred. That's one example of how I've received information.

Near-death and out-of-body experiences are another way that people unintentionally gain noetic information. One participant described an experience during a group meditation:

I felt myself speeding through space as though on a roller coaster. Suddenly I heard a loud pop and my soul and consciousness left my body. Floating in absolute silence, warm, safe space I knew immediately that this is how we die, that I was connected to everything and that I had left my ego. I saw a faint golden light way off in the distance and then heard a voice call my name and waves of energy carrying my name came over me like waves of an ocean, indescribable love washed over me and then suddenly I was back in my body in the meditation room.

In summary, how people engage with access information or energy varied and occurred both intentional and spontaneous ways. For intentional experiences, there was no one way that people prepare themselves, although some oft-cited practices were meditation, setting intentions, breathwork, and dreams. These engagement practices could induce a state of awareness that is different from the normal waking awareness level.

**Theme 2: Ways of knowing**

The ways of knowing theme was the largest response category and had five subthemes: Intuitive, Embodied, Sensorial, Emotional, and Direct.

***Intuitive***

The most common way of knowing is what many would call intuition. Intuition is the ability to understand something immediately from an instinctive feeling or without conscious reasoning. Intuitive codes with the number of participants who mentioned them were: intuitive-just knowing (184), just arrives/pops into head/download (104), and intuitive, not otherwise specified (13). Typical example quotes for this way of knowing are: “There is simply a knowing,” “I just know it,” “It just pops into my head,” “It was like the information was just downloaded,” “Something that just lights a light bulb of aha,” and “It is like I just receive these packets of information about a person or situation.”

This intuitive experience is often described as ineffable and without rational or logical reasons for knowing the information, such as, “I have no idea how, but I know these things.” The experience is mental. The information is described as just arriving in the mind fully formed, without effort, and often spontaneously.

**Embodied**

Participants also described knowing things through their bodies. Similar to how people who experience intuition said, “I just know it,” people with embodied experiences said, “I just feel it” (106). It is not something they know with their mind but with their body; they viscerally feel the information. Such as, “When I was a child, I would play a game with my sisters about when would dad come through the door after work, and I would always be spot on, as I could just feel it or sense it.”

Other sub-codes for embodied were: embodied not otherwise specified (39), and bodily reactions or side effects to noetic experiences such as adverse emotional reactions (47), or physical reactions (36), or side effects in general (21).

Specific sensations in the body were also shared as a way participants knew they were accessing information or energy beyond our conventional notions of time and space, such as goosebumps or chills (24), hot (70) or cold (25), pain (26) or discomfort (16), or “gut” sensations (32). Some people felt tingles, vibrations, electric or magnetic sensations in their bodies (85). Such as, “I know when I have a spirit around me because I get a tingling sensation between my left hip and knee,” or “I always get an electrical “buzz” down my neck and spine. A vibrational feeling or a surge of energy like a shock wave.”

Participants also frequently mentioned changes in temperature. Heat was often also mentioned specifically in the hands and in relation to healing intention. “My hands get really hot and tingly when I'm in the flow.”

Whilst doing Reiki and other forms of healing on myself and others, I can feel the energy flowing through my hands and my hands can get quite warm. The healing energy always seems to go to where it is most required. The energy can also be channelled into the past or the future and in distance healing. I have had this confirmed by people I have worked on. The energy is not coming from me but through me. Anyone can access this energy. I also get goosebumps too when I'm in places of high energy like crop circles, Stonehenge, etc.

Many people also described feeling goosebumps or chills, not from being cold, but as a signal that they were receiving some truth or should continue along the path discussed. Such as, “I definitely act on my goosebumps and make a decision. So if I come up with an idea and immediately feel goosebumps, I will follow that idea,” and “Having goosebumps is for me a sign that I am on the right path, or should do exactly what I was just thinking of.”

Participants also mentioned the typical “gut hunch” experience where they have a physical sensation in their stomach. This gut hunch gives them a sign or some information. Such as, “I get nauseous and a heavy feeling in my stomach when something wrong with those I love. I think of people, then up to 10 minutes later they will call me.” Another participant said:

I have the capacity to use energy in a healing sense, or felt sensation. But I feel it is heavily intertwined to the information aspects, energy for me is felt only after I see an image of the healing. It can be felt alone but it seems to only be in a sort of “gut” reaction to people either in a loving sense or if I feel uncomfortable for one reason or another. To which I would examine the situation further with my “informative” senses, almost to confirm.

Other participants mentioned physical sensations along with intuitive and embodied type of experiences.

I have thoughts come into my head. They are like remembering something but they are memories of the future and not of the past. They just appear in my thought stream but they have an emotional component. Like a warm sense in my solar plexus that tells me these thoughts are not like ordinary thoughts or imaginings. They are precognitive knowings. I feel a definite sense of knowing they are the truth.

Some people described actually feeling, in their own bodies, the pain others are experiencing. Interestingly, in some cases, when the person realizes that it is not theirs, the pain or discomfort will dissipate.

I was riding in a car with a good friend who had injured her knee in a skiing accident. I knew about the injury, but she didn't go into detail about it. We drove about fifty miles on a scenic drive and then stopped for coffee. I could barely move my right leg, and my right knee hurt like crazy. I asked her if her injured leg was bothering her and which leg it was. She said yes, and it was the right knee. When I realized that it was her physical discomfort I was feeling, the pain and the stiffness in my own leg and knee disappeared.

I am never sick and rarely ever have aches or pains. When I am around others and suddenly have pain, I know it is not mine. I've checked this with people too. I asked one friend, “Steve, you have a pain behind your left eye, radiating through the temple, and back through under your ear.” He said, “Yes, it's been hurting me all day.”

***Sensorial***

The previous embodied experiences are generalized over the participant's whole body. Other descriptions could be related to the traditional five senses but with heightened perception. Sub-themes for each sense included:

*Seeing.* Many people described “seeing” inner visions or images in their minds (171).

Sometimes the information is a flash of visual awareness, sometimes instantaneously almost before the question is formed. One time my husband misplaced his keys. He was looking all over for them. All of a sudden, I “saw” them hidden under some papers in the bottom drawer of his office desk. When I gave him this information, he returned to work and went exactly to the spot I had described. Sure enough, he found the keys hidden from view under a bunch of papers in his desk drawer.

I experience pictures/visions of something and these (for what I know) are happening in real-time. When they come, I am wide awake and could be having a conversation with someone and a picture or image flashes in my head or in front of my eyes.

Outer seeing is a less common experience (58), described as observing things in one's surroundings that are not ordinarily visible with physical eyes. One might see lights and/or colors around others, recently passed loved ones, or other non-physical beings, like guides and angels.

I met my grandmother (who passed away) at midnight like we meet any one personally. She used to love me very much. I was not present with her in her last days. I was in room alone, sleeping, at midnight she appeared in front of me with a crystal white clothes and called me and showed her everlasting love to me. After some time she disappeared.

*Hearing.* Hearing an inner voice was commonly reported in our study (151). Many of these messages happened spontaneously to protect from danger.

I have had experiences of hearing a voice warning me not to go to a certain address in my working service. As I was traveling to patients' homes, I would occasionally “hear” a voice that was not my own, give me a short important message to follow, like, “Don't go there now.” I followed that message. When I went there later, the police were there and had arrested someone for robbing people in the lobby of that building.

I can hear information spoken to me in words, phrases and sentences and I can sometimes obtain detailed information such as names, dates and locations.

Not all messages are related to danger or decisions that need to be made. Other participants expressed receiving insight, creative ideas, and additional useful information through their inner voice.

*Touch.* People described two types of noetic experiences involving touch (40). People would touch an item and gain information about its owner or other information that one would not usually know.

I can pick up information by touch alone, and this has taken me back in time through the life events experienced by that object to its creation. For example I can trace the life of a carved crystal right back to its formation in the earth and receive images of all the places, people, and situations that crystal has been through, including the vibration of carving.

The other occurred in healing settings, where people working in healing therapies would touch a person and get information about that person's emotions, life situation, and past traumas.

Another aspect of touch is feeling perceived non-physical beings. Some people seem to be more sensitive to feeling non-physical beings. They may not see or hear them but can feel the non-physical beings touching them.

*Smelling and tasting.* Super smell (25) and taste (11) senses were not as common but were reported.

One time, I suddenly smelled my niece's vomit—very intense and disgusting. I then searched my home for signs that someone was sick only to find that no one was. At the same time, I received a knowing that this smell was connected to my sister who lives a great distance away, across many states. Sure enough, about twenty minutes later, I received a phone call from my sister explaining that she'd been at the movies when her daughter got sick and she was in the restroom holding back her hair while she vomited into the toilet.

Experiences of taste not related to the physical are even more unusual but also happen. In one example, someone described how they have a way of “tasting” energy. When they contemplate future ideas, they get a taste in the back of their throat that gives them information about their decision. Other sensorial responses were about hearing external sounds that others could not hear (53), vestibular/movement changes (10), and sensorial experiences not otherwise specified (23) that the participant attributed to a noetic experience.

**Emotional**

Another way people know things is through emotions (57). Empathy is a standard human capacity. Understanding or feeling what another person is going through from their perspective is part of being a loving, caring human. However, many people experience empathy on a different level beyond this ordinary capacity. They can actually feel other people's emotions directly.

I am an empath. I feel energy viscerally in my body. For example, when others feel strong emotions, particularly grief. I feel the same emotions within myself. I can feel in my body before someone will cry.

I don't like being in large crowds as I am so aware of everyone else's feelings. Sometimes I will draw from the collective energies in the crowd to give me the strength to just be there and sustain the bombardment of the emotions.

**Direct**

Another way of knowing is direct, where the information is perceived to come from something, someone, or somewhere else (90). Many also mentioned specific sources to the information, such as animals (40), the environment (34), deceased people (74), and other non-physical beings (e.g., spirits, spirit guides) (112). Participants described deceased loved ones appearing in dreams and giving messages. The loved one gave a specific message to the dreaming person or a message to pass on to another person.

I only have two experiences of death of someone really close to me, my boyfriend and my mom. My boyfriend talked to me once (without voice, transfer of thought forms only if that makes sense) while I was meditating. My mom visited once in my vivid dream. Both messages conveyed that they are okay.

Pictures falling, radios turning on, objects being displaced, and other physical manifestations are credited to deceased loved ones too. Spiritual guides, angels, spirits, and other entities are also reported to give direct information that a person needs.

I often will ask my “beings” for guidance - it may be a yes/no to something (e.g., is it good to visit...today?). I generally will often get a very clear yes or no to things and I have found that if I follow this, things go well but if I go against this, which I very rarely do now - (it's usually just that I forgot to ask and check in...) that things do not go well. I use this for guidance around many things - planning work things, food and what is good for me at times, social things etc.

I believe that I am helped and developed in my sleep by these loving beings, my ancestors, also what people now call guides or angels. What they are called doesn't matter to me, only that they are loving beings.

Numerous people expressed being able to directly get information from others' thoughts or intentions and even animals' minds. “Energetically, I feel deceased people and animals around me. I also have animals energetically ‘talk’ to me.” People also said they got information directly from living people (23) or communication with others (19). For example, one participant had to make a decision. They internally asked the Universe what they should do. Within ten minutes, someone came up to them and said something relevant to the choice they needed to make.

People feel they know things through symbols that appear to them (56). The repetition of a particular or unusual symbol may keep appearing that has meaning to the person.

Another way I appear to receive information is via things that happen in my external world, such as the same symbol being shown to me over and over. Last year when my mother was in the hospital, the number nineteen kept presenting itself repeatedly. My mother even noticed the strangeness associated with all the coincidences surrounding this number. It was as if someone was trying to tell me something.

People directly knew energy and information as it moved through their body too.

Sometimes they received direct information from the energy (51) and information (63). Other times, they would need to interpret what the energy or information meant. Others mentioned picking up information from the environment like, “Picking up on energy when entering a room.”

**Theme 3: Types of information**

Participants often commented on the specific content that they accessed or received through their ways of knowing. Knowing information about the future, such as an event that was going to occur or a warning about an impending danger, was very common (219). Another large information category was about the nature of reality and the person's place in it (124). Knowing information about distant locations (51) that the person never visited was another common type of information. Information about an item or object was mentioned (43). Self-awareness insight, such as information about personal events the person was dealing with (31), was another area. Information about other people was also noted in general (17), and also others' thoughts (114), emotions (97), physical sensations (44), and facts (134) about the person that they could not possibly know through traditional means. Gaining information about their or others' health was also noted (12).

**Theme 4: Ways of affecting**

The ways of knowing theme revolved around more passive or receptive activities. The ways of affecting theme is about the effect the information or energy has on the world. These encompass participants' responses about how they influence their world, such as through healing, decision making, influencing others and the environment with their mind or intentions. Participants mentioned communicating the information they received to others (68), often in the context of helping them (99).

***Healing***

Healing in general (46), healing others (152), healing self (72), using healing systems (58), with Reiki (55) being the most commonly named system, were commonly expressed noetic experiences of healing with “intention,” “energy,” “thoughts,” or “the mind.” Some of these examples are quite remarkable and unexplainable by normal means. Such as, “I healed my stage IV cancer by bringing healing light/energy into me (from the Creator) and visualizing it healing my tumor, by aligning my EM [electromagnetic] energy with the planet, and by creating a state of grateful energy.” Another participant stated, “I was near someone who had a bruise or illness and described it. I have watched as their bruise or illness dissipates, and I suddenly have the bruise in the same spot or illness. (I avoid sick people).” The most remarkable response recounted the healing of a child's brain tumor.

Everything is connected. I was part of a healing circle at church. A toddler had an inoperable brain tumor. The whole church connected by touching and focusing on healing an innocent child. I SAW the energy, like a golden web, lines extending from one person to another to another, then flowing into the child. 2 weeks later, his doctor's were EXTREMELY confused....no tumor, no cancer. Just a healthy child.

**Decision making**

Another widespread comment—in fact, one of the top ten most common comments—was that the information supported the participants in making decisions in their lives (196). For example, people remarked that they could receive information about upcoming events in their lives and then change their behavior based on that information. “I use this energy in my daily life for making decisions” “I take it as important guidance and use it regularly in my life … I make life choices and decisions according to this information.”

Many people described how powerful the noetic experiences were for their self-growth. The information or energy they received supported a deeper self-awareness, introspection, or reflection for the respondents (144) or changing their perceptions of their self-identity (33).

**Influencing others**

Some people also experience their thoughts affecting someone else or influencing others somehow (91), like being in the same dream with another person, influencing someone within a dream, and synchronistic connections across time and space.

In 1986, I was stuck in a job that I didn't like as a bookkeeper. What I really wanted was to work as a bookkeeper with an accountant whom I'd worked with a couple of years previously. I did not contact her directly. Instead, I placed a thought “out there” (wherever “there” is) that she should contact me. Four days later, she called and offered me a job.

The above experience is a similar concept to visualization or manifestation that was frequently mentioned. Many people talked about visualizing something they wanted to have happen and then observing it manifest at some point in the immediate future (38).

I don't formally access it. I have an ability to manifest. Something creative pops into my head... that office, that home, that car, this life, this degree, this course... I must be carrying it around in my subconscious. At some point, I feel this sort of “click” in my chest in my core and I say to myself, “oh it's going to happen,” and then it does.

This happened about 10 years ago. I was taking a walk in my neighborhood in the autumn. I'd been walking for about an hour, and I was getting cold. As I turned for home, I wished I'd worn a hat and made a mental note to buy one soon. I even thought about the kind of knit beanie I'd like to get. I did not intentionally “put the thought out.” But when I got to my house there was a clean knit beanie placed on the tree stump at the end of the walkway exactly like the one I was thinking about. These kinds of occurrences happen with some frequency. I wish I'd kept a journal of them!

One important thing to note is that others' influence can be used with ill will or harmful intent. A few people said they used their intention to harm others (21). For example, one person wrote, “I sent negative energy to him and he got a flat tire, which can happen to anyone, but this was on a brand new motorcycle with brand new tires. Coincidence? Maybe, but I believe it was me.”

**Influencing systems, objects, the environment**

Ninety-four participants mentioned using the mind or mental intention to influence systems, objects, or the environment. On participant noted:

As an experiment, I wanted free electricity at my house. I went to sleep with the intention to stop the meter from turning. In a dream that night, I was able to put my mind inside the meter. When I awoke, I remembered the dream instantly and was very excited. I got dressed barely containing my excitement, ran outside, and saw that the meter was stopped. I asked my kids to turn the central air [conditioning] on and then off again [to see if the electric meter would respond], and there was no movement on the meter. I had free electricity for two months except for a base-charge minimum. I got worried that the electric company would come out and change the meter, so I went outside and put my hand on it. I asked my son to turn the central air off and on again. I removed my hand, and the meter was turning again.

**Theme 5: Ways of expressing**

Because two of the survey questions specifically asked how people expressed the information and energy they access through noetic experiences, a theme emerged aligned with this inquiry. The amount of text people wrote for these two questions was much less compared to the two accessing questions. Expression mainly was described as communicating it with others in various ways such as talking, writing, social media, and other interactions with others (197). The other significant way of expressing was through creative practices like dance (21), music (40), visual art and drawing (77), journaling or writing for self-use (151), and other various creative practices (43), such as, “I paint, write, dance, and make movies.”

## Discussion

In summary, this study evaluated participants' free-response observations of their noetic experiences. The study focused on participants accessing and expressing information and energy beyond conventional notions of time and space, and thus, beyond the traditional five senses. The study's objective was to better understand the phenomenology of noetic experiences and its variation. This study's findings support the idea that noetic experiences are widespread and are experienced in specific and yet variable ways.

Five themes emerged from the thematic analysis of the dataset: 1) ways of engagement, 2) ways of knowing, 3) ways of affecting, 4) ways of expressing, and 5) types of information. Participants detailed a variety of ways in which they experience these themes in their lives. Generally, participants ascribed significant meaning and positive benefit from the experiences for themselves and others.

### Ways of engagement

Interestingly, the survey questions did not specifically ask participants how they achieved states conducive to having noetic experiences. Despite this, most participants mentioned the situation in which the noetic experiences occurred, whether intentional or spontaneous. Of the intentional experiences, numerous tools were described that allowed the participant to achieve an altered state of consciousness conducive to the noetic experience. For example, many people mentioned meditation as one such gateway tool. Being a meditator or being in a meditative state is one of the strongest predictors for noetic experiences ( Roney-Dougal, 2015; Vieten *et al.*, 2018). Multiple meditation electroencephalography (EEG) and neuroimaging studies demonstrate state and trait changes in the brain ( Wahbeh, Sagher, *et al.*, 2018; Fox *et al.*, 2014, 2016). Sacred rituals and practices that induce altered states of consciousness and are meant to inspire access to inner knowing are ubiquitous in numerous cultures worldwide ( Krippner, 2000). Dreams and sleep were another commonly mentioned way participants engaged in accessing noetic information intentionally and spontaneously. Numerous laboratory studies have shown that people can access noetic information while asleep ( Sherwood & Roe 2003; Storm *et al.*, 2017; Storm & Rock, 2015).

Spontaneous experiences were also pervasive. These unintentional experiences align with models, such as FSMT and PMIR, that propose noetic experiences as an innate aspect of ourselves and serve a functional role. Often the information received was protective or warning of danger as well. Participants expressed multiple pathways to the noetic experience with varying levels of control for their occurrence.

One final finding to note about engagement methods was the profoundly spiritual nature of the experiences that people described. People frequently experience “the noetic” as interconnectedness or a connection to a force or power greater than their individual selves (e.g., Higher Self, God, Spirit, Source, Universe, Interconnected Field, Higher Consciousness, Divine).

### Ways of knowing

The participants described multiple ways of knowing, including intuitive, embodied, sensorial, emotional, and direct. Each of these has been previously observed in laboratory and real-world settings.

**Intuitive**

Participants just know something is true about people, places, or situations that could not be known or inferred by rational thought with intuitive knowing. As William James noted, this state of understanding comes with a feeling of authority on the knowledge. A typical participant statement was, “I just know it.” Numerous controlled experiments have explored the nature of general intuitive knowing ( Cardeña, 2018; Cardeña, Palmer, & Marcusson-Clavertz, 2015; Radin, 2013; Schwartz, 2010).

**Embodied**

Our participants described a few aspects of embodied knowing. The most common was a pure knowingness that they perceived in the body rather than in the mind. Others have noted that our bodies are physically sensitive to others' mental intentions ( Radin & Pierce, 2015). Meta-analyses of dozens of independently conducted experiments of this type have shown that when a sender directs their attention toward a distant person, it does influence the receiver's physiological state ( Schmidt, 2012, 2015; Schmidt *et al.*, 2004). Telesomatic experiences, described as typically unhealthy or harmful physical symptoms shared by people at a distance, have also been evaluated ( Dossey, 1994, 1995, 2008b, 2016; Mann & Jaye, 2007; Neppe, 1984; Schwarz, 1967, 1973).

**Sensorial**

Inner vision, voice, and touch are well-evaluated noetic experiences. Inner vision, or what some people call remote viewing, has been formally evaluated in multiple studies and meta-analyses ( Baptista *et al.*, 2015; Cardeña, 2018; Dunne & Jahn, 2003; Milton, 1997). Inner vision has also demonstrated verifiable and practical applications, including the famous military Star Gate program run from 1973 – 1995 ( May & Marwaha, 2018a, 2018b), predicting the stock market, futures or other financial market information, sport event outcomes, locations of missing persons or criminal cases, and finding unknown archaeological sites ( Schwartz, De Mattei, & Smith, 2019; Schwartz, 2019; Kolodziejzyk, 2013).

Inner voices have been recorded since ancient times. Apparently, Socrates once heeded a warning an inner voice gave him and avoided being trampled by a herd of pigs ( Hastings, 1991, p. 119). Researcher Alfred Alschuler reviewed historical figures who heard inner voices and found 150 individuals, including Martin Luther King Jr. and Saint Theresa ( Hastings, 1991, p. 121). There is growing interest among mental health researchers who are reframing these experiences as normal instead of being signs of pathology ( Lee, 2020; National Hearing Voices Network, 2021; Powers & Quagan, 2021; Powers III *et al.*, 2017; Richardson, 2018).

Another sensorial way of knowing that participants described was the ability to touch an object and gain knowledge from it or learn about people who owned it or were near it, which is often referred to as psychometry. While much research was conducted in the late 1800s and early 1900s ( Roll, 2004), little research has been conducted recently ( Baker *et al.*, 2017; Parra & Argibay, 2007, 2009a, 2009b).

**Emotional**

In this study, the noetic correlate of inner emotional knowing goes beyond ordinary empathy. Participants explained experiences of knowing another's emotions in detail, without the usual interpersonal signals or clues we associate with empathy. Researchers have studied the prevalence of this noetic emotional connect, its potential evolutionary advantage, and how it relates to other noetic experiences ( Aron *et al.*, 2012; Aron & Aron, 1997; Irwin, 2017).

**Direct**

One main noetic source participants mentioned was deceased people. Purported communication with the deceased has been well-studied in the context of mental mediumship ( Beischel & Zingrone, 2015; Temple and Harper 2009; Rock *et al.*, 2020; Sarraf, Woodley, & Tressoldi, 2020; Braude 2003; Fontana 2005), including multiple studies using triple-blind methods demonstrating that mediums can obtain verifiably correct information about deceased people that they could not have known through traditional means ( Beischel & Schwartz, 2007; Beischel *et al.*, 2015; Delorme *et al.*, 2013, 2018). Also, people reporting contact with the dead is a globally widespread phenomenon, with 25-53% of people reporting that they had contact with the dead ( Greeley, 1975, 1987; Haraldsson & Houtkooper, 1991; Pew Research Center, 2009).

### Types of information

Participants expressed receiving many different types of information via noetic means. Numerous laboratory studies demonstrate this experience, showing that people can know the future both consciously ( Storm, Tressoldi, & Di Risio, 2012; Honorton, Ferrari, & Hansen, 2018) and unconsciously ( Mossbridge & Radin, 2017; Storm & Tressoldi, 2020). Others have explored knowing the future in everyday life ( Alvarado, 2008; Dossey, 2008a, 2009). Knowing other people's thoughts, also known as telepathy, has also been rigorously studied with many studies demonstrating telepathic email, text messages, and telephone connections ( Sheldrake *et al.*, 2015; Sheldrake & Avraamides, 2009; Sheldrake & Beeharee, 2016; Sheldrake & Smart, 2005). Other studies in the laboratory have found clear evidence for telepathy using mild sensory deprivation techniques called the ganzfeld effect ( Cardeña 2018; Storm & Tressoldi 2020; Baptista, Derakhshani, & Tressoldi, 2015; Storm, Tressoldi, & Di Risio, 2010).

### Ways of affecting

Participants detailed many ways that noetic experiences and information had affected their lives. Numerous experiments where positive intention is directed at humans, animals, plants, and cells, have found small but significant positive results ( Roe *et al.*, 2015). Energy medicine techniques such as Therapeutic Touch and Reiki have also been shown to positively affect conditions like pain, cancer, mental health symptoms, and hypertension ( Jain *et al.*, 2015; Rao *et al.*, 2016; Yount *et al.*, 2021). Extraordinary case studies of spontaneous remissions have also been noted ( O'Regan & Hirshberg, 1993).

Using noetic experiences for decision-making, self-healing, awareness, or introspection has also been noted in numerous studies where positive impact is imparted on people's lives ( Ellison & Fan 2008; Griffiths *et al.*, 2008; Kennedy & Kanthamani, 1995; Moreira-Almeida & Cardeña, 2011; Negro Jr, Palladino-Negro, & Louzã, 2002; Richards, 1991; Wahbeh, Radin, *et al.*, 2018; Wahbeh *et al.*, 2019; Wahbeh, McDermott, & Sagher, 2018; Wahbeh & Butzer, 2020; Sagher, Butzer, & Wahbeh, 2019).

Mind-matter interactions have also been rigorously studied in the laboratory, where people have directed their intention to a random system and produced effects ( Schmidt, 1974; Varvoglis & Bancel, 2015; Jahn *et al.,* 2007; Dunne & Jahn, 1992; Bosch, Steinkamp, & Boller, 2006; Radin *et al.*, 2006). We can also see these effects in field-experiments ( Nelson, 1997; Nelson *et al.*, 1996; Radin, 2018) and global experiments of indirect intention effects ( Nelson, 2015).

### Limitations

There are several limitations of this study that should be considered when reviewing the results. First, the study has all the limitations inherent in qualitative research, such as, subjective reporting, difficulty ascertaining causality, errors in memory recall, and limits in generalizability based on a specific sample pool. Also, we asked four particular questions which necessarily informed the answers given. Regardless, the results brought great insight into people's phenomenological experience of the noetic.

## Conclusion

In conclusion, this study found five major themes regarding participants' perceptions of the noetic experience encompassing how they engage in the experiences, their states of knowledge and understanding, and their capacity for influencing the world around them. Future research will include investigating the nuances of these themes and establishing standardized methods for evaluating them, then informing curricula and therapies to support people in these experiences. Ultimately, these noetic experiences impart positive benefits for the person. We anticipate that persistent efforts to break taboos on noetic topics will bring light to these common and usually beneficial experiences.

## Data availability

### Underlying data

Figshare: Noetic Signature Qualitative Survey supplemental data. https://doi.org/10.6084/m9.figshare.14449704.

This project contains the following underlying data:

• Raw data file which includes participants' free response answers.

### Extended data

Figshare: Noetic Signature Qualitative Survey supplemental data. https://doi.org/10.6084/m9.figshare.14449704.

This project contains the following supportive material:

• Survey file which includes specific questions asked of participants.

Data are available under the terms of the Creative Commons Attribution 0 International license (CC 0).
