# Supplementary material for: Qualitative analysis of first-person accounts of noetic experiences
Source: F1000Res. 2022 Mar 11;10:497. Originally published 2021 Jun 25. [Version 3] doi: 10.12688/f1000research.52957.3 (PMC9364752; doi:10.12688/f1000research.52957.3)
Supplement: Peer review history

# Peer review: Reviewer response for version 3

**DOI:** 10.5256/f1000research.121498.r127137

**Published:** 2022-04-08

**Authors:** Jacob Jolij

**Affiliations:** 1 Heymans Institute for Psychological Research, University of Groningen, Groningen, The Netherlands

My concerns have been addressed.

Is the work clearly and accurately presented and does it cite the current literature?

Partly

If applicable, is the statistical analysis and its interpretation appropriate?

Yes

Are all the source data underlying the results available to ensure full reproducibility?

Partly

Is the study design appropriate and is the work technically sound?

Yes

Are the conclusions drawn adequately supported by the results?

Yes

Are sufficient details of methods and analysis provided to allow replication by others?

Yes

Reviewer Expertise:

Psychology, parapsychology, human research ethics

I confirm that I have read this submission and believe that I have an appropriate level of expertise to confirm that it is of an acceptable scientific standard.

# Peer review: Reviewer response for version 2

**DOI:** 10.5256/f1000research.74237.r115513

**Published:** 2022-01-20

**Authors:** Jacob Jolij

**Affiliations:** 1 Heymans Institute for Psychological Research, University of Groningen, Groningen, The Netherlands

The authors present the results of a large scale survey about exceptional experiences. The data are mainly presented 'as is', without too much interpretation, which is entirely appropriate for a paper like this. However, I am afraid I cannot approve the paper in its present version. There are two main reason for this:

 1) The paper lacks an appropriate discussion of the main stream literature.

 2) There is a very serious problem with ethics and privacy regarding the dataset.

 Both of these problems, however, can be addressed, and once they have been fixed I believe this paper to make an excellent and highly valuable contribution to the literature on exceptional experiences. I would like to emphasize that in particular point 2 is very important and without addressing this in full I cannot approve this paper. 

 I detail my two points of criticism below:

 1) The discussion of the reported experiences is very strongly biased toward the literature on parapsychology. To some extent this is understandable given the background of the authors, but it neglects the large and rapidly growing body of literature in mainstream psychology on transformative experiences, and the growing realization that such experiences can have a massive impact on, for example, mental health. I feel the authors should at least include a brief discussion of the main stream literature on exceptional/transformative experiences before this paper can be approved.

 2) The data is provided with this paper and can be downloaded without restrictions. Undoubtedly this is inspired by the open science and open data movement, but in this case it is entirely inappropriate. I applaud the authors for making their data available, but in this case the data should have been put in a restricted access repository. Although the data do not contain any direct identifiers under the US HIPAA regulations, the questionnaire lists very sensitive data, such as race, income, and unabridged qualitative reports of exceptional experiences, which are of course highly identifiable.

 Under the EU GDPR, this dataset would be considered to be non-anonymized. Moreover, the dataset contains sensitive data: as the authors note in their article, the content of noetic or exceptional experiences is often considered to be very private and related to people's personal faith and/or life philosophies. Under the GDPR, this is considered to be a special category of data on which can only be collected for the purpose of scientific research. The same applies to race, and financial status. As this dataset is published openly, the authors cannot guarantee that this data is exclusively used for scientific research, so for GDPR this creates a problem.

 Of course, the authors are based in the US, but the problems with anonymization create several problems. First, EU researchers cannot use this data without more information regarding the informed consent, as EU researchers are bound by the GDPR. However, a more serious issue is that the questionnaire includes data from several EU countries and the UK. The legal rights of these participants have been violated by the public sharing of this dataset, even though these participants and authors may not realize this.

 Fortunately, this can be easily remedied by immediately removing the data from the public repository and move it to a repository like Dataverse, or another repository with a restricted access policy.

Is the work clearly and accurately presented and does it cite the current literature?

Partly

If applicable, is the statistical analysis and its interpretation appropriate?

Yes

Are all the source data underlying the results available to ensure full reproducibility?

Partly

Is the study design appropriate and is the work technically sound?

Yes

Are the conclusions drawn adequately supported by the results?

Yes

Are sufficient details of methods and analysis provided to allow replication by others?

Yes

Reviewer Expertise:

Psychology, parapsychology, human research ethics

I confirm that I have read this submission and believe that I have an appropriate level of expertise to state that I do not consider it to be of an acceptable scientific standard, for reasons outlined above.

# Response

**Published:** 2022-02-17

**Authors:** Helané Wahbeh

**Affiliations:** Oregon Health & Science University, USA

Thank you for your thoughtful review of our manuscript. Based on your comments, we have made changes to the manuscript that we feel strengthened it. Please see the manuscript changes outlined in italics text below each comment summary. We hope these changes have met your conditions to approve the manuscript. 

 1) The discussion of the reported experiences is very strongly biased toward the literature on parapsychology...  I feel the authors should at least include a brief discussion of the main stream literature on exceptional/transformative experiences before this paper can be approved.

*-Thank you for this suggestion, which has helped place our manuscript in a broader context. We have added to the introduction a brief discussion of the growing body of mainstream psychology literature on transformative experiences and how this relates to noetic experiences.*

 2) The data is provided with this paper and can be downloaded without restrictions...

 Fortunately, this can be easily remedied by immediately removing the data from the public repository and move it to a repository like Dataverse, or another repository with a restricted access policy.

*-Thank you for your feedback on data accessibility. Yes, we had included because of the open science and open data movement. According to our US standards and Institutional Review Board requirements, this dataset is anonymous. We understand that may not be the case in the EU because of GDPR standards. We have now removed the dataset from the public record. It is now on Figshare with a permanent embargo. This means that the public cannot access it without requesting permission. Here is the new link https://doi.org/ 10.6084/m9.figshare.19157867*

# Peer review: Reviewer response for version 2

**DOI:** 10.5256/f1000research.74237.r92572

**Published:** 2021-08-31

**Authors:** Everton Maraldi

**Affiliations:** 1 Post-Graduate Program on Religious Studies, Pontifical Catholic University of São Paulo, São Paulo, Brazil

I have now read the authors' responses and the revised article. I have no other comments to make. I am ok with the changes made.

Is the work clearly and accurately presented and does it cite the current literature?

Yes

If applicable, is the statistical analysis and its interpretation appropriate?

Yes

Are all the source data underlying the results available to ensure full reproducibility?

Yes

Is the study design appropriate and is the work technically sound?

Yes

Are the conclusions drawn adequately supported by the results?

Yes

Are sufficient details of methods and analysis provided to allow replication by others?

Yes

Reviewer Expertise:

NA

I confirm that I have read this submission and believe that I have an appropriate level of expertise to confirm that it is of an acceptable scientific standard.

# Peer review: Reviewer response for version 1

**DOI:** 10.5256/f1000research.56294.r88280

**Published:** 2021-07-06

**Authors:** Everton Maraldi

**Affiliations:** 1 Post-Graduate Program on Religious Studies, Pontifical Catholic University of São Paulo, São Paulo, Brazil

This is an interesting study that discusses topics of relevance to the investigation of experiences variously termed anomalous, psychic, noetic or spiritual. The methodological approach is sound, and the paper provides a clear presentation of the qualitative data. While this paper should be of interest to readers of F1000Reseach and is certainly worthy of publication, I do have a number of comments that require attention: 

- The definition of noetic experiences as states of knowledge or insight may well apply to telepathy, clairvoyance, and precognition but does not seem to fit psychokinesis or anomalous physical phenomena... This should be further clarified. 
- What do you mean by energy? Did you provide a definition of this term in the questionnaire? If you decided to use this term because of its common usage among experiencers, you should make this more explicit in the paper and provide additional references to back up your decision. For example, were the ʺanecdotal expressions that people perceive noetic experiences as information and energyʺ collected as part of a previous study? Also, you should acknowledge the possibility that some individuals may prefer not to use the term energy to define their own experiences and that this might have influenced their responses to (or decision to answer) the survey. 
- It would be important to ask participants about their self-reported spirituality and religious or philosophical affiliation. Many noetic experiences are appraised as religious or spiritual (see, for example, the work of William James). This limitation should be acknowledged and adequately justified, especially in view of the finding that participants reported the ´profoundly spiritual nature of the experiences´.
- Even though this a qualitative study, the use of tables to highlight the frequency of codes and themes would help improve the description of the findings, especially considering the sample size. 
- The discussion section combines phenomenological and ontological approaches to the study of noetic experiences, but the differences and approximations between these approaches merit further discussion. For example, you discuss the findings of the survey with reference to several laboratory studies of psi. However, it is important to explain that the evidence obtained in the laboratory is not always generalizable to psi experiences outside the lab (and vice-versa). Also, I would expect some discussion of the possible biases involved in more anecdotal reports, as well as the potential contribution of your study to the improvement of laboratory research on psi (for example, in what concerns ecological validity). 

Is the work clearly and accurately presented and does it cite the current literature?

Yes

If applicable, is the statistical analysis and its interpretation appropriate?

Yes

Are all the source data underlying the results available to ensure full reproducibility?

Yes

Is the study design appropriate and is the work technically sound?

Yes

Are the conclusions drawn adequately supported by the results?

Yes

Are sufficient details of methods and analysis provided to allow replication by others?

Yes

Reviewer Expertise:

Psychology of religious experiences; spirituality, religion and health; dissociation and dissociative disorders; anomalous experiences; paranormal beliefs.

I confirm that I have read this submission and believe that I have an appropriate level of expertise to confirm that it is of an acceptable scientific standard, however I have significant reservations, as outlined above.

# Response

**Published:** 2021-08-06

**Authors:** Helané Wahbeh

**Affiliations:** Oregon Health & Science University, USA

Thank you for your thoughtful consideration of our manuscript. We have edited the manuscript in response to your comments which we feel has strengthened it. Please see our responses below each comment.

 The definition of noetic experiences as states of knowledge or insight may well apply to telepathy, clairvoyance, and precognition but does not seem to fit psychokinesis or anomalous physical phenomena... This should be further clarified.

**Response:** We have added some language in the manuscript about expressive versus receptive psi to address this comment.

 What do you mean by energy? Did you provide a definition of this term in the questionnaire? If you decided to use this term because of its common usage among experiencers, you should make this more explicit in the paper and provide additional references to back up your decision. For example, were the ʺanecdotal expressions that people perceive noetic experiences as information and energyʺ collected as part of a previous study? Also, you should acknowledge the possibility that some individuals may prefer not to use the term energy to define their own experiences and that this might have influenced their responses to (or decision to answer) the survey.

**Response: **We included a definition of energy in the survey participants completed. The full text for this is included in the Supplemental Data. We have added some information on this in the main manuscript text with a reference. In the limitations section, we have also acknowledged that the use of the word information or energy may have influenced their responses or decision to complete the survey.

 It would be important to ask participants about their self-reported spirituality and religious or philosophical affiliation. Many noetic experiences are appraised as religious or spiritual (see, for example, the work of William James). This limitation should be acknowledged and adequately justified, especially in view of the finding that participants reported the ´profoundly spiritual nature of the experiences´.

**Response: **For this anonymous qualitative study, we collected limited demographic information about the participants and did not include their religious and spiritual backgrounds. We have added this in the limitations section.

 Even though this a qualitative study, the use of tables to highlight the frequency of codes and themes would help improve the description of the findings, especially considering the sample size.

**Response:** We have added four tables with the subthemes and their frequencies. Numerical counts have been removed from the text.

 The discussion section combines phenomenological and ontological approaches to the study of noetic experiences, but the differences and approximations between these approaches merit further discussion. For example, you discuss the findings of the survey with reference to several laboratory studies of psi. However, it is important to explain that the evidence obtained in the laboratory is not always generalizable to psi experiences outside the lab (and vice-versa).

**Response: **We have added language about this concept at the beginning of the discussion section.

 Also, I would expect some discussion of the possible biases involved in more anecdotal reports,

**Response:** This has been made clearer in the limitations section.

 as well as the potential contribution of your study to the improvement of laboratory research on psi (for example, in what concerns ecological validity).

**Response:** The potential contribution of the various themes has been highlighted in various sections of the discussion.
